# Antioxidant and anti-inflammatory effects of *Schisandra* and *Paeonia* extracts in the treatment of asthma

**DOI:** 10.3892/etm.2014.1948

**Published:** 2014-09-04

**Authors:** XIA CHEN, YI HUANG, JING FENG, XIAO-FANG JIANG, WAN-FEI XIAO, XIAO-XI CHEN

**Affiliations:** Department of Pediatrics, The 324th Hospital of PLA, Chongqing 400020, P.R. China

**Keywords:** *Schisandra*, *Paeonia*, asthma, antioxidant, anti-inflammatory

## Abstract

The aim of the present study was to investigate the antioxidant and anti-inflammatory effects of *Paeonia* and *Schisandra* extracts in asthmatic rats. An ethanol extraction method was used to prepare the *Schisandra* and *Paeonia* extracts, and the levels of hydroxyl radical, total antioxidant activity and total phenolic content were detected. The rats were divided into three groups: Treatment (group A), model (group B) and control (group C). The treatment group received traditional Chinese antiasthmatic medicine (mixed extract, 2 ml/day) for 10 days. Levels of malondialdehyde (MDA), Cu-Zn-superoxide dismutase (SOD) and glutathione peroxidase (GSH-Px) were detected in the serum, while interleukin (IL)-4, IL-6, interferon (IFN)-γ, IL-13 and IL-22 levels were analyzed in the serum, bronchoalveolar lavage fluid and lung tissue homogenates of the three groups. In addition, pathological changes of the tracheal tissues were observed via biopsies and the NF-κB p65 level was measured in the lung tissue using immunohistochemistry. Total antioxidant activity, hydroxyl radical levels and total phenolic content in the mixed herbal extracts were higher than those in the single herbal extracts. At day 5 following the treatment, the number of eosinophils was significantly reduced in the tracheal tissues. At day 10 following the treatment, the mucosa was significantly repaired. *In vivo* antioxidant levels revealed that the serum and erythrocyte SOD activity and GSH-Px were higher in group A as compared with group B, while the level of MDA in group A was lower than that in group B (P<0.05). The levels of serum and erythrocyte SOD activity and GSH-Px in group B were lower than those in group C, while the level of MDA in group B was higher than that in group C (P<0.05). IL-4, IL-6 and IL-13 levels in the serum, bronchoalveolar lavage fluid and lung tissue in group A were not significantly different from those in group B (P>0.05). However, IFN-γ levels in group A significantly increased as compared with the level in group B, while IL-22 levels decreased significantly in group A as compared with group B (P<0.05). IL-4, IL-6, IL-13 and IL-22 levels in the lung tissue, bronchoalveolar lavage fluid and serum in group B were significantly higher than those in group C. In addition, the IFN-γ level decreased significantly in group B as compared with the level in group C (P<0.05). Immunohistochemical analysis revealed that the protein expression of NF-κB p65 in group A was significantly lower compared with group B (P<0.05). Therefore, *Paeonia* and *Schisandra* extracts may be used to treat asthma through their *in vivo* antioxidant and anti-inflammatory effects.

## Introduction

Asthma is a lung disease characterized by chronic inflammation of the airways (1). An oxidant/antioxidant imbalance in asthma is one of the important mechanisms underlying the inflammation process. Therefore, supplementation of antioxidants in order to maintain the oxidant/antioxidant balance can significantly reduce airway inflammation and improve ventilation and airway remodeling. In traditional Chinese medicine, there are a large number of natural antioxidant substances, including phenolic acids and saponins (2). Over the years, the use of traditional Chinese medicine in the treatment of asthma and other respiratory inflammatory diseases has exhibited unique effects when compared with conventional treatments, including the use of inhaled corticosteroids and oral leukotriene receptor antagonists (3). Chinese medicine not only produces an anti-inflammatory response, it also exhibits immunoregulatory effects and reduces the release of oxygen free radicals (4). Xiao-qing-long-tang is a well-known drug that has been shown to be effective for the treatment of coughs and asthma and has a far-reaching impact in the history of Chinese medicine development. *Paeonia* and *Schisandra* extracts are administered as adjuvant drugs in xiao-qing-long-tang; this combination has exhibited considerable success. However, the anti-inflammatory mechanisms of the two herbs in the treatment of asthma remain unclear. Previous studies have demonstrated that *Paeonia* contain saponins, while *Schisandra* contain other antioxidants, including schisandrin B. However, the effects of *Paeonia* and *Schisandra* in an *in vivo* model of asthma remain unclear (5–7). Thus, the present study aimed to investigate the underlying mechanisms of *Paeonia* and *Schisandra* in the treatment of asthma.

## Materials and methods

### Ethics statement

This study was approved by the Ethics Committee of the 324th Hospital of PLA (Chongqing, China). Animal care guidelines were strictly followed for all experimental procedures in the study.

### Extract preparation

*Schisandra* and peony original drugs were extracted from Chinese medicine solution, in accordance with the ratio of 1:1 using ethanol extraction methods. The reflux time was 5 h and the extracts were placed in a sealed container at 4°C.

### Preparation of asthmatic rats

Rats (n=200) were purchased from the Third Military Medical University Laboratory Animal Center (Chongqing, China) and were maintained under specific pathogen free conditions. The rats were randomly divided into treatment (group A), asthma (group B) and saline control groups (group C). For the rats in the asthma and treatment groups, 200 mg aluminum hydroxide, (Hebei Xinhua Pharmaceutical Company, Shijiazhuang, China) 1 mg ovalbumin (Hebei Xinhua Pharmaceutical Company) and 0.2 ml saline (0.9%; Hebei Xinhua Pharmaceutical Company) were administered subcutaneously. Once every two days, an additional subcutaneous injection of 0.2 ml inactivated *Bordetella pertussis* (Qilu Pharmaceutical Co., Ltd. Jinan, China) was administered. Following sensitization, 2% ovalbumin inhalation was applied for stimulation once a day for one week. In the control group, normal saline was used to replace the subcutaneous injection of antigen solution. In the asthma and treatment groups, the rats exhibited symptoms such as sneezing, scratching heads, shortness of breath, abdominal muscle contraction, agitation, hair removal and hair thinning. The treatment group received 2 ml herbal extracts via daily gavage for 10 days. Blood samples (2 ml) were centrifuged for analysis prior to treatment and at day 5 and 10 following treatment in the rats of the three groups. At day 10 following treatment, the rats were sacrificed by cervical dislocation and the trachea tissues were removed for hematoxylin and eosin staining and NF-κB activity analysis in the three groups.

### In vitro hydroxyl radical, total antioxidant activity and total phenolic content determination in the herbal extracts

A total antioxidant activity assay was performed with the herbal extracts using a kit from the Nanjing Jiancheng Bioengineering Institute (Nanjing, China). Determining the hydroxyl radical suppression of the herbal extracts was conducted according to the Fenton reaction principle. The procedure was performed using a hydroxyl radical assay kit, according to the manufacturer’s instructions (Nanjing Jiancheng Bioengineering Institute). Determination of the total phenolic content in the herbal extracts was measured using Lowry reagents (Sigma-Aldrich, St. Louis, MO, USA).

### Serum malondialdehyde (MDA), superoxide dismutase (SOD) and glutathione peroxidase (GSH-Px) measurement

These parameters were measured using ELISA kits, according to the manufacturer’s instructions (Biovalue Co., Ltd., Shanghai, China.

### Immunohistochemistry

Paraffin-embedded sections, 4-μm thick, were obtained from the lung tissues. Following dewaxing, xylene and graded ethanol dehydration was performed. Hydrogen peroxide (3%) was used to inactivate endogenous peroxidase antigens. Following serum sealing for 30 min, primary antibodies against NF-κB p65 (Beyotime Institute of Biotechnology, Haimen, China) were added and incubated for 1 h. Staining was then performed using a 3,3′-diaminobenzidine staining kit (Biotek Co. Ltd. Beijing, China). Phosphate-buffered saline was used in the negative control group instead of primary antibody. A positive reaction was indicated by brown staining in the cytoplasm and nuclei. In three high magnification fields, the positive rate was calculated as the ratio of positive cells/total cells.

### Statistical analysis

Statistical analysis was performed using the Statistical Package for Social Sciences computer software (version 17.0; SPSS, Inc., Chicago, IL, USA) for Windows. Analysis was conducted using the χ^2^ test and the analysis of variance method. P<0.05 was considered to indicate a statistically significant difference.

## Results

### Histology

**Figure 1:**
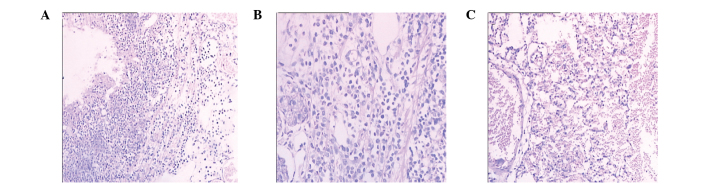
Immunohistochemistry images of the (A) control group at day 0 and the (B) control and (C) treatment (traditional Chinese medicine) groups at day 10 following treatment (magnification, ×100).

As shown in Fig. 1A, the tracheal mucosa and columnar epithelium underwent exfoliation and evident eosinophil infiltration was observed prior to treatment. As shown in Fig. 1B, infiltration of a large number of eosinophils, neutrophils and lymphocytes occurred at day 10 following saline treatment. However, in the traditional Chinese medicine treatment group, the trachea cilia and muscle layer were normal, and marked eosinophil infiltration was not observed, as shown in Fig. 1C.

### In vitro antioxidant activity

As shown in Table I, the total antioxidant activity, hydroxyl free radical level and total phenolic content in the mixed extract were higher than those in the single herb extracts.

### Determination of erythrocyte antioxidant activity

As shown in Table II, serum and erythrocyte SOD activity and GSH-Px levels in group A were higher than those in group B, while the level of MDA in group A was lower than that in group B (P<0.05). Serum and erythrocyte SOD activity and GSH-Px levels in group B were lower than those in group C, and the MDA level in group B was higher than that in group C (P<0.05).

### Determination of cytokine levels

IL-4, IL-6 and IL-13 levels in the serum (Table III), bronchoalveolar lavage fluid (Table IV) and lung tissue homogenates (Table V) were not significantly different between groups A and B (P>0.05). However, IFN-γ levels significantly increased in group A as compared with group B, and IL-22 levels decreased significantly (P<0.05). The levels of IL-4, IL-6, IL-13 and IL-22 in the lung tissue, bronchoalveolar lavage fluid and serum of group B were significantly higher than those in group C, while the IFN-γ level decreased significantly (P<0.05).

## Discussion

When asthma occurs, airway inflammation results in increased levels of proinflammatory cytokines, NADPH oxidase and reactive oxygen species or reactive nitrogen radicals. Under normal circumstances, antioxidants in the lung tissue can remove small amounts of reactive oxygen species or reactive nitrogen radicals. Only an excessive amount of reactive oxygen species or reactive nitrogen radicals causes damage to airway proteins, lipids and DNA, resulting in airway inflammation, airway hyperresponsiveness, airway microvascular hyperpermeability and airway mucus hypersecretion. However, continued inflammation increases the levels of apoptosis and necrosis, which is followed by lung tissue oxidative damage (8). Therefore, oxidative stress is an important mechanism in the pathogenesis of asthma. To reduce oxidative stress or increase the antioxidant function, decreasing airway eosinophil infiltration, mucus secretion, airway hyperresponsiveness and changes in airway remodeling is required. Antioxidant supplementation has become a new method for the treatment of asthma. Chinese traditional medicine, including phenols, polysaccharides, alkaloids and saponins, are recognized as strong antioxidants that exhibit a free radical scavenging effect (9,10). Water and ethanol extracts containing these Chinese medicinal ingredients have been shown to exhibit strong anti-inflammatory and anticytotoxic effects. *In vitro* experiments have demonstrated that these extracts can inhibit nitric oxide (NO) and tumor necrosis factor-α production (11). The antioxidant activity of Chinese medicinal raw herbs is low (12), however, a water or ethanol extract of these Chinese medicinal raw materials exhibits significantly increased antioxidant activity. Therefore, extracting the active ingredients of Chinese medicinal raw materials is becoming increasing popular in the development of Chinese medicine. In our preliminary study, an ethanol extract reflux time of 5 h was shown to produce the highest antioxidant activity *in vitro*.

The traditional Chinese medicinal formula, xiao-qing-long-tang, has a strong therapeutic effect on asthma and has been shown to regulate protein secretion in airway club cells (13). In addition, the formula has been shown to reduce the proportion of eosinophils in the serum and thus, the levels of IL-5 and histamine release. The medicine can stabilize mast cell membranes, inhibit mast cell degranulation and also inhibit endothelin-1 levels and NO synthesis (14). *Schisandra* species, that are in xiao-qing-long-tang, are a type of superior medicine that have been used in China for almost one thousand years. *Schisandra* has been shown to exhibit antioxidant, cough and asthma inhibiting and liver function protective effects (15). Modern pharmacological studies have shown that *Schisandra* exhibits biological and pharmacological effects predominantly due to lignans (16). These lignans include schisandrin, deoxyschisandrin, schisandrin B, schisandrin C, schisandrol B, schisantherrin A, schisantherrin B and gomisin A. Levels of lignans in *Schisandra* water extract can reach ~99% (17). *Paeonia* also has a very long history of use in China and can significantly reduce eosinophil chemokine expression and secretion. In addition, *Paeonia* can inhibit the migration of eosinophils in A549 culture medium and the activation of NF-κB. *Paeonia* species have a therapeutic effect on asthma (18). A study of 44 types of traditional Chinese medicine revealed that there were relatively high phenolic and flavonoid levels in *Akebia*, *Aster* and water or ethanol extract of *Paeonia*. Therefore, *Paeonia* is one of the three traditional Chinese medicines with the highest anti-inflammatory and antioxidant activities (19). In the present study, following the joint use of *Paeonia* and *Schisandra*, the total antioxidant activity was 1.963±0.211 mmol/g, hydroxyl radical inhibition was 368.55±1.27 U/ml and the total phenolic content was 2055.32±4.65 μg/ml, which was significantly improved as compared with the single herb extracts. Levels of oxidative stress are closely associated with airway inflammation (20).

The level of oxidative stress *in vivo* is reflected through the oxide levels and antioxidant capacity in the body. During an asthma attack, the vitality of inflammatory cells increases, the function of airway epithelial cilia is impaired, mucus secretion increases and a large number of free radicals are produced. Active substances, including SOD, GSH-Px and vitamin C and E, are involved in the pathophysiological process of asthma (21). SOD exists in the cytoplasm and lysosome and can transform O_2_ into H_2_O; GSH-Px is also involved in this process. Free radicals induce body injury mainly through lipid peroxidation, following the reaction of unsaturated fatty acids, ethylene, pentane and MDA (22). Thus, following lipid injury of the cells, the end products increase. Therefore, asthma treatment should focus on increasing SOD and GSH-Px enzyme activity and reducing the increase in oxidation products. During an asthma attack, MDA levels increase, while GSH-Px levels decrease (23).

In the present study, serum and erythrocyte SOD activity and GSH-Px levels were lower in group B than in group C, while MDA levels in group B were higher than in group C (P<0.05). These results further illustrate the effect of oxidative stress on asthma. *Paeonia* and *Schisandra* extracts were used in the treatment of asthmatic rats, and the levels of serum and erythrocyte SOD activity and GSH-Px in the treatment group were found to be higher than those in group B (asthma group). In addition, the MDA level was lower than that in group B (P<0.05). Therefore, *Paeonia* and *Schisandra* extracts can significantly increase the antioxidant levels and reduce oxidative damage.

Cytokines, inflammatory cells and inflammatory mediators interact with each other, resulting in the infiltration of a large number of airway eosinophils and CD4^+^ T cells, as well as mucus hypersecretion, airway hyperresponsiveness, airway remodeling and increased IgE production. An imbalance in the cytokine levels produced by Th1, Th2 and Th17 cells leads to asthma. Therefore, regulating cytokine levels is the focus for asthma treatment (24). T lymphocytes play a major regulatory role in asthmatic airway inflammation, with Thl cell subsets secreting IFN-γ, which mediates immune responses and delayed-type hypersensitivity. Th2 cell subsets primarily produce IL-4, IL-5 and IL-13, which may induce the production and aggregation of eosinophils, as well as stimulate B cells to produce IgE and mediate humoral immune responses and engineering-type hypersensitivity. IL-22 is a proinflammatory cytokine that exhibits anti-inflammatory activity. IL-22 belongs to the IL-10 cytokine family and has two functional receptors, IL-22R1 and IL-22R2 (25). IL-22 may be activated by a variety of signaling pathways, the most important being the signal transducer and activator of transcription 3 pathway (26). In an antigen-sensitized rat model, IL-22 levels significantly increased (27). Neutralizing IL-22 antibodies can increase the production of Th2 cytokines, causing eosinophil infiltration and airway hyperresponsiveness (28). By contrast, IL-22 is a protective agent for airway inflammation, and recombinant IL-22 can inhibit eosinophilic airway inflammation and the production of Th2 cytokines (29,30). IL-22 also inhibits IFN-γ-inducible expression of proinflammatory cytokines and human airway epithelial cell adhesion (31). Therefore, the function of IL-22 is closely associated with the external environment and changes with the body disease state.

In conclusion, this study investigated the antioxidant and anti-inflammatory effects of *Schisandra* and *Paeonia* extract and showed that these effects could be beneficial in alleviating the asthmatic condition. Therefore, *Schisandra* and *Paeonia* extract could be used in the future treatment of asthma.

## Figures and Tables

**Table I:** Antioxidant activity of the herbal extracts.

Parameter	*Schisandra* extract	Herbaceous peony extract	Mixed extract
Total antioxidant activity (mmol/g)	1.482±0.115^a^	0.556±0.031^a^	1.963±0.211
Hydroxyl free radical (U/ml)	316.24±1.46^a^	89.09±3.76^a^	368.55±1.27
Total phenol content (μg/ml)	1138.26±6.53^a^	1253.28±5.91^a^	2055.32±4.65

Total antioxidant activity was determined using the ABTS method.

a P<0.05 vs. the mixed extract.

**Table II:** Serum antioxidant activity.

Parameter	Group A	Group B	Group C
SOD activity (U/l)	0.5±0.012^a^	0.33±0.025^a^	0.62±0.025
GSH-Px (μmol/l)	3166±252^a^	1650±125^a^	3250±203
MDA (μmol/l)	125.93±13.05^a^	511.334±15.363^a^	324.162±16.472

a P<0.05 vs. group C.

SOD, superoxide dismutase; GSH-Px, glutathione peroxidase; MDA, malondialdehyde.

**Table III:** Cytokine levels in the serum.

Cytokine (pg/ml)	Group A	Group B	Group C
IL-4	115.06±30.53^a^	173.78±32.85^a^	123.67±12.55
IL-6	997.92±145.31^a^	2005.23±125.62^a^	759.54±102.47
IFN-γ	1434.07±115.63^a^	766.83±84.05^a^	1226.14±125.01
IL-13	766.54±35.24^a^	859.32±55.08^a^	583.28±39.12
IL-22	50.20±11.31^a^	123.47±20.25^a^	62.35±15.18

a P<0.05 vs. group C.

IL, interleukin; IFN, interferon.

**Table IV:** Cytokine levels in the bronchoalveolar lavage fluid.

Cytokine (pg/ml)	Group A	Group B	Group C
IL-4	40.81±8.46^a^	39.75±8.35^a^	28.09±7.66
IL-6	83.52±12.65^a^	187.19±13.62^a^	51.09±10.21
IFN-γ	259.96±25.31^a^	148.69±19.34^a^	235.61±22.04
IL-13	46.64±11.22^a^	45.32±10.07^a^	28.96±9.62
IL-22	92.68±15.24^a^	125.96±20.34^a^	82.95±10.21

a P<0.05 vs. group C.

IL, interleukin; IFN, interferon.

**Table V:** Cytokine levels in the lung homogenates.

Cytokine (pg/ml)	Group A	Group B	Group C
IL-4	43.29±3.06^a^	47.35±5.33^a^	30.57±4.31
IL-6	89.62±8.69^a^	187.19±10.08^a^	59.61±9.64
IFN-γ	389.14±25.93^a^	202.08±29.18^a^	354.35±35.77
IL-13	47.26±8.41^a^	46.86±6.22^a^	25.75±5.93
IL-22	88.56±12.03^a^	125.37±15.84^a^	67.42±8.67

a P<0.05 vs. group C.

IL, interleukin; IFN, interferon.
